# 

**Published:** 1920-10-09

**Authors:** 


### Recent Official Publications.

(To be obtained at H.M. Stationery Office, Imperial House, Kingsway, W.C. 2. The prices include postage.)

Women and Young Persons (Employment in Lead Processes Bill). 1½d.

Hygiene of Food and Drink. Syllabus of Lessons for Use in Schools, and Notes for the Assistance of Teachers. 3d.

Medical Research Committee. Reports upon Investigations in the United Kingdom of Dysentery Cases received from the Eastern Mediterranean. 7d.

### Gifts and Bequests.

The Ingham Infirmary, South Shields, has been left by the late Mr. Walter Marshall Chadwick, City of Bayonne, New Jersey, U.S.A., $50,000, free of tax (approximately £14,250). The executors wish that a ward may be named after Mr. Chadwick.

Mr. George Harding, of Oxton, Cheshire, has left £200 each to the following Liverpool charities: The Royal Southern Hospital, the David Lewis Northern Hospital, the Seamen's Orphanage, Birkenhead Borough Hospital, Birkenhead Children's Hospital, the Liverpool Children's Hospital, Heswall, and the Birkenhead Maternity Hospital.

King George's Fund for Sailors have made a grant of £2,500 for capital expenditure to King George V. Merchant Seamen's Hospital at Malta.

Mr. H. W. Edwards, of Newton Abbot, Devon, left £200 to the Birmingham General Hospital and £150 each to the Birmingham and Midland Hospital for Women and Children and the Newton Abbot Hospital.

The Arsenal Football Club have decided to endow a bed in the Great Northern Hospital, the donation amounting to one thousand guineas, which will be paid in annual instalments.

Dr. Charles Workman, of Glasgow, Examiner in Pathology to the Royal Faculty of Physicians and Surgeons, Glasgow, formerly medical officer at the Belfast Fever Hospital and the Ulster Hospital for Children, left personal estate in the United Kingdom valued at £96,797.

Dr. Joseph Needham, of 34 King's Avenue, Clapham Park, and Harley Street, formerly Professor of Anatomy in the University of Aberdeen, medical officer of the Board of Education, left £10,100.

### Matrons' and Sisters' Appointments.

TORBAY HOSPITAL AND EYE INFIRMARY, TORQUAY.—Miss Norah Meredith has been appointed night sister. She was trained at the Royal Victoria and West Hampshire Hospital, where she was later holiday sister, and has since done private nursing.

BATTERSEA BOROUGH COUNCIL MATERNITY HOME, WANDSWORTH COMMON.—Miss Charlotte Dickson has been appointed matron. She was trained at the West London and Queen Charlotte's Hospitals, and has held the post of sister at Great Ormond Street, Hampstead, General, Samaritan Free, and Queen's (for Children) Hospitals.

GRAMPIAN SANATORIUM, KINGUSSIE, INVERNESS.—Miss Bessie F. Robertson has been appointed sister. She was trained at the Royal Infirmary, Dundee, and has been sister of the tubercle wards at the Longmore Hospital, Edinburgh.

GREENWICH UNION INFIRMARY.—Miss Annie McEllin has been appointed first assistant matron. She was trained at Preccot Infirmary, and has been assistant matron at the Poor-Law Institution, Wigan, and previously night superintendent and ward sister at the Billinge Military Hospital. Miss McEllin holds the certificate of the Central Midwives Board.

SEVERALLS MENTAL HOSPITAL, COLCHESTER.—Miss E. M. Cuthbert has been appointed matron. She was trained at the Southport General Infirmary, has been matron and housekeeper at the Ryhope Mental Hospital, Sunderland, and previously assistant matron of the Edinburgh Royal Asylum and night superintendent at the Montrose Royal Asylum.

INFECTIOUS DISEASES HOSPITAL, BARRY.—Miss S. E. Ascott has been appointed matron. She was trained at the Manchester Royal Infirmary and the Park Fever Hospital, Lewisham. She has been home sister and assistant matron of the County Sanatorium, Hawkmoor, sister of the Northern Convalescent Fever Hospital, Winchmore Hill, and staff nurse in the Territorial Nursing Service at the 4th London General Hospital and the 42nd General Hospital, Salonica.

ROYAL ALEXANDRA HOSPITAL, RHYL.—Miss Eleanor Johnson has been appointed ward sister. She was trained at the Liverpool Children's Infirmary, and was later sister of the children's wards at the North Staffordshire Hospital, and medical sister at the Royal Hospital for Sick Children, Glasgow.

ROYAL VICTORIA HOSPITAL, DOVER.—Miss E. Riley has been appointed night sister at this hospital, where she was trained.

GENERAL HOSPITAL, BRIDGWATER.—Miss Elsie B. Edman has been appointed sister. She was trained at the London Hospital, where she was later sister, has been sister-in-charge at Woodcote Military Hospital, Reading, has worked at Bath War Hospital, and been holiday sister at the Boscombe Hospital, Bournemouth.

CLAYTON HOSPITAL, WAKEFIELD.—Miss Alexandrina Linton, at present night sister, has been appointed assistant matron. She was trained at Oldham Royal Infirmary, was later attached to the Scottish Women's Hospitals under Dr. Elsie Inglis' Unit, and served in Serbia. Miss Elizabeth Shervington has been appointed night sister. She was trained at Manchester Royal Infirmary, and has served as sister and night sister in the Territorial Force Nursing Service.

FEVER HOSPITAL, BIRKENHEAD.—Miss M. Roberts has been appointed night superintendent. She was trained at the Blackburn Royal Infirmary and the Borough Hospital, Ryde.

ELLESMERE PORT AND DISTRICT COTTAGE HOSPITAL.—Miss Lilian M. Jeans, A.R.R.C., has been appointed matron. She was trained at Charing Cross Hospital and joined Q.A.I.M.N.S.R. in August 1914, serving as theatre sister at the Royal Herbert Hospital, Woolwich until 1915. She then served in Egypt and France, and was mentioned in despatches. Miss Jeans, who is a member of the College of Nursing, holds the certificate of the Central Midwives Board.

ISOLATION HOSPITAL, MUSWELL HILL.—Miss Florence Pollard and Miss N. Strachan have been appointed sisters. Miss Pollard was trained at St. George's Infirmary, and the Western Fever Hospital, was later staff nurse at the South-Eastern Fever Hospital, and sister at Clare Hall Sanatorium, Barnet. Miss Strachan was trained at the Royal Victoria Hospital, Dundee, and the City Hospital, Edinburgh, and was later sister at the War Hospital, Bath, and the Poor-Law Hospital, Dundee.

ROYAL INFIRMARY, LANCASTER.—Miss Elizabeth Goold has been appointed home sister. She was trained at King's College Hospital, and after doing private nursing joined the Q.A.I.M.N.S.R., and served abroad as sister for four years; latterly she has filled the post of home sister and housekeeping sister at the Radcliffe Infirmary, Oxford.

ST. CHAD'S HOSPITAL, BIRMINGHAM.—Miss Jessie Buttfield has been appointed sister. She was trained at Lincoln County Hospital, was ward sister, night sister, and theatre sister at Bute Hospital, Bedford, and later night superintendent and ward sister at the North Lonsdale Hospital, Barrow-in-Furness.

### The Book Market.

Cassell and Co., Ltd.: "Diathermy in Medical and Surgical Practice." By Claude Saberton, M.B. 7s. 6d. net.

H. K. Lewis and Co., Ltd.: "The Extra Pharmacopœia." By Martindale and Westcott. Vol. I. Seventeenth Edition. 27s. net.

Cassell and Co., Ltd.: "The Assessment of Physical Fitness." By Georges Dreyer, C.B.E., M.A., M.D., and G. F. Hanson. 10s. net.

John Murray: "Rhoda Drake." By C. H. Dudley Ward, D.S.O., M.C. 7s. net.

